# *In vivo* real-time red blood cell migration and microcirculation flow synergy imaging-surveyed thrombolytic therapy with iron-oxide complexes

**DOI:** 10.1016/j.mtbio.2022.100408

**Published:** 2022-08-26

**Authors:** Fei Ye, Bei Zhang, Lige Qiu, Yunrui Zhang, Yang Zhang, Jian Zhang, Qingliang Zhao, Ligong Lu, Zhenlin Zhang

**Affiliations:** a Zhuhai Interventional Medical Center, Zhuhai Precision Medical Center, Zhuhai People's Hospital (Zhuhai Hospital Affiliated with Jinan University), Zhuhai, 519000, PR China; b State Key Laboratory of Molecular Vaccinology and Molecular Diagnostics, Center for Molecular Imaging and Translational Medicine, School of Public Health, Shenzhen Research Institute of Xiamen University, Xiamen University, Xiamen, 361102, PR China; c The Sixth Affiliated Hospital of Guangzhou Medical University, Qingyuan People's Hospital, Department of Biomedical Engineering, School of Basic Medical Sciences, Guangzhou Medical University, Guangzhou, 511436, PR China; d Department of Pharmacy, Zhuhai People's Hospital (Zhuhai Hospital Affiliated with Jinan University), Zhuhai, 519000, PR China

**Keywords:** Thrombosis, Thrombolysis, Red blood cell, Capillary microscopy, Microcirculation flow imaging

## Abstract

Nanotherapeutics as a nascent method has attracted widely interest on the treatment of thrombosis. However, due to the limited temporal and spatial resolution of conventional imaging modalities, the dynamic visualization the thrombogenesis and evaluation of the effect of thrombolytic drugs are facing severely difficulties *in vivo*. In addition, the development of high targeting, short circulation time, and small size thrombolysis nanotherapeutics agents requires further research. Herein, we report a synergy imaging modality that combining a label-free capillary microscopy and laser speckle microcirculation imaging, which realized dynamic visualization of single red blood cell migration and large-field dynamic blood flow. In this work, we investigated the red blood cells migration and blood flow velocity response before and after treated through introducing a functional nano-thrombolytics, iron-oxide complexes coated urokinase (IPN@UK) on an orthotopic animal model *in vivo*. The functionalized IPN@UK nanocomposites exhibited outstanding thrombolysis effect. Significantly, whole-course changes, including red blood cell activity, complex thrombolytic therapeutics, were well surveilled and evaluated using dual-modality combining imaging strategy. These results show this synergy imaging strategy not only can achieve multiscale non-invasive visualization of dynamic thrombus events in real-time, but also can quantify hemodynamics information of thrombus. Our study demonstrates the potential of this synergy imaging method, which for early detection of thrombus, evaluation of the effect of drug thrombolysis, developing the thrombolytic drugs, and imaging-guide thrombolytic therapy in living systems.

## Graphical abstract

**Figure:**
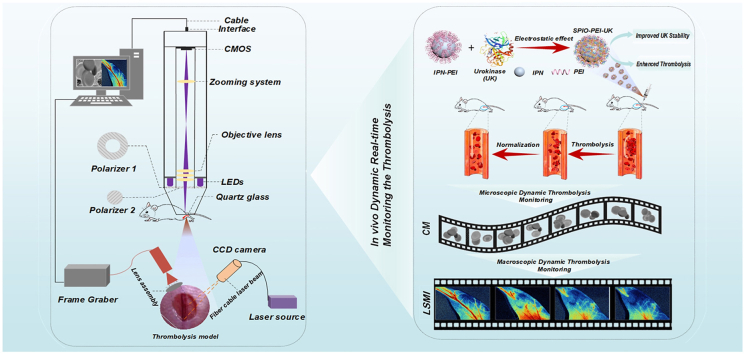

## 1 Introduction

Thrombosis is the most common pathology of the three major cardiovascular diseases, ischemic heart disease, stroke, and venous thromboembolism, and is also one of the leading causes of death and disability worldwide [[1], [2], [3]]. Thrombosis can be divided into arterial thrombosis (AT) and venous thrombosis (VT). Generally, venous thrombi happen in deep veins most often in the hind legs or forearms, this process named the deep vein thrombosis [4]. Once the thrombus occurs, it can lead to blockage or absolute occlusion of blood circulation system, which may produce ischemic stroke, acute myocardial infarction, pulmonary vascular embolism, and other crucial events [5]. Current clinical thrombolytic therapy primary is injection of thrombolytics, such as urokinase (uPA), streptokinase (SK) and recombinant plasminogen agents (rt-PA) for thrombolysis therapeutics [6,7]. As we all known, all thrombi possess a comparable quantity of fibrin (35–43%), making this a target of commonly for these thrombolytics. Thus, the plasmin can be stimulated activation after treated with these therapeutics, and then the thrombus can be degraded or dissolved. Despite these therapeutics has a high efficacy in treatment the antithrombotic, these protein based thrombolytics suﬀer from a short half-life, allergic reactions, or inactivation [[8], [9], [10]]. Additionally, due to all therapeutics through activation organism own haemostatic systems, this system balanced could be destroy during inhibiting thrombus formation process. Unfortunately, these processes might could cause hemorrhage in body or sites and other severe thrombus complications. Furthermore, the thrombolysis eﬀect is limited considerably by the poor targeting ability and low enrichment efficiency [9]. In summary, the current therapeutics of thrombosis is extremely limited to the advanced situation, led to a narrow therapeutic window, making it difficult to afford effective treatment. Therefore, there is a clinical need to improve thrombosis treatment by developing a new method that can accurately locate and qualitatively diagnose thrombosis at an early stage.

Recently, nanotherapeutics as a nascent method has attracted widely interest on the treatment of thrombosis ﬁeld [[11], [12], [13]]. The unique physical and chemical peculiarities of nanoparticles (NPs) offer the feasibility for its structure, function, and targeting efficacy can be controlled in specific application. However, practical limitations concerned with this approach such as therapeutic dose discrepancy, circulation time and large size. Thus, these concerns should be taken into consideration when developing rational design of nanocrystallization thrombolytics. Another most important considerations when exploiting a novel thrombolytics is the intended imaging technique. In the process of thrombosis, red blood cells (RBC), white blood cells (WBC), platelets, coagulation, thrombus markers, and blood flow conditions play an important role [[14], [15], [16], [17], [18]]. The changes in RBCs that affect thrombosis and thrombolysis, include RBC counts or hematocrits and qualitative changes, such as deformability and aggregation. In the process of thrombosis, the aggregation of RBCs is accompanied by the interruption of RBCs migration. In the process of thrombolysis, the aggregated RBCs and hemoglobin are dissolved, which will be accompanied by the recovery of blood flow and the gradual recovery of RBCs migration. On the other hand, in the case of thromboembolisms and thrombolysis, the blood flow velocity and dynamic thrombolysis response for the detection of thrombi are peculiarly crucial in decreasing mortality and morbidity. Many imaging modalities have been used for thrombus imaging, including ultrasound imaging (US) [19,20], magnetic resonance imaging (MRI) [21,22], and positron emission tomography (PET) [23,24]. However, they all suffer from limited spatial resolutions and are only suitable for advanced thrombosis [25]. Besides, they cannot provide high contrast imaging of thrombosis and thrombolytic process, which limits the diagnosis of early thrombosis and the development of thrombolytics. In recent years, new imaging modalities have been introduced into the detection of thrombus, such as photoacoustic imaging (PAI) [25]. PAI can penetrate biological tissues, and it has the advantages of non-invasiveness and high resolution. However, high-resolution photoacoustic microscopy based on point scanning mode is difficult to realize dynamic monitoring of blood flow and micro thrombus. Optical techniques have high spatial and temporal resolution and can resolve subcellular structures in a wide range of applications [[26], [27], [28]]. Dynamic blood flow velocity (BLV) imaging of microcirculation may shine a new light on possible pathophysiological mechanisms of various thrombus diseases [29]. Furthermore, *in vivo* real-time, temporal-spatial resolution and dynamic visualization these responses can provide new strategies to therapeutic thrombosis.

Orthogonal polarization spectral (OPS) and side-stream dark field (SDF) imaging techniques use unique absorption properties of RBCs and crossed polarizers to achieve high temporal and spatial resolution of label-free capillary imaging and show good potential of RBCs imaging *in vivo*, and they are widely used in the nailfold and sublingual microcirculation imaging at present [[30], [31], [32], [33], [34], [35]]. Due to the advantages of SDF and OPS for *in vivo* imaging of microcirculations and RBCs, SDF and OPS have potential applications for high-resolution imaging of thrombus. However, the imaging field of view (FOV) of the SDF and OPS are limited. Laser speckle microcirculation imaging (LSMI) is a promising technology for modern theranostic nanomedicine application, which has attracted great research interest from various research fields. It's able to directly non-invasively monitor blood flow and vascular structure, at high spatial and temporal resolution in larger FOV *in vivo* [36,37], which is crucial to investigate the management of thrombus. As a result, combining the OBS/SDF with LSMI advantages, which can not only potentially offer high resolution visualization of single cell, but also provide the hemodynamics information of thrombus in the larger view. In other words, their complementarity that are great significance for assessing thrombogenesis and thrombolytic procedures, exploiting thrombolytic drugs and drug delivery systems for thrombus treatment.

**Scheme 1:**
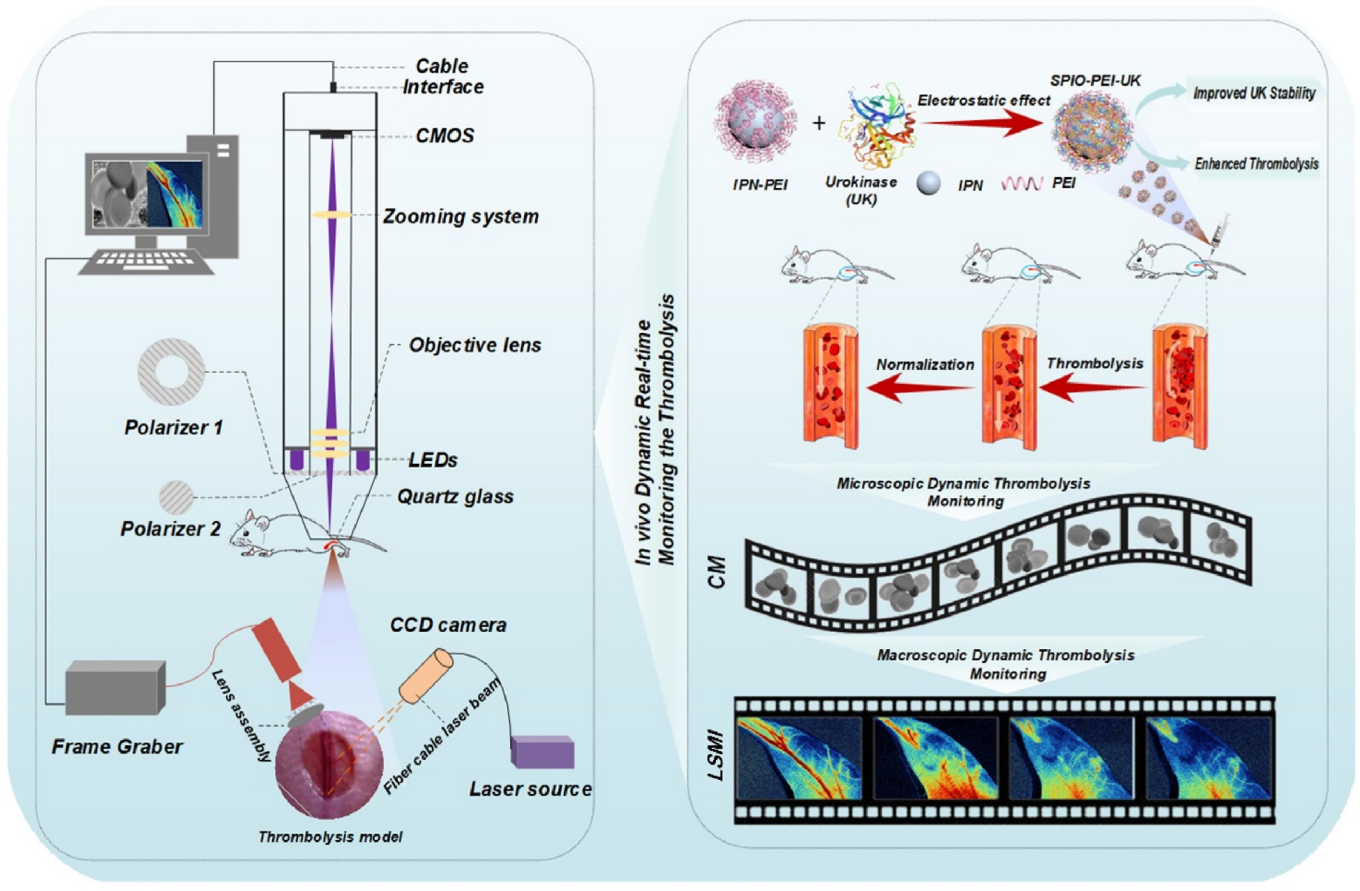
Schematic diagram of experimental setup and materials. The IPN@UK nanocomposites were prepared by mixing an appropriate amount of solution containing various amount of IPN with UK ligands in PBS (pH 7.4), and then incubated at room temperature for 20 ​min for further investigations. IPN@UK nano-thrombolytics nanomaterials were injected to the vein of mouse model, and the whole process of thrombolysis by IPN@UK nano-thrombolytics nanomaterials was monitored by synergy CM and LSMI. Combining the advantages of CM with LSMI can potentially provide not only cell-level resolution visualization of single RBCs, but also hemodynamic information of the thrombus in a larger field of view. UK: urokinase, IPN: iron-oxide nanoparticles, PEI: alkyl-PEI2k-PEG2k, CM: Capillary microscopy, LSMI: Laser speckle microcirculation imaging.

In this work, we reported a label-free capillary microscopy (CM) based on SDF technology that can perform cell-level resolution imaging of thrombus, as well as real-time dynamic imaging of RBC migration at single-cell level and observation of the flow response of the microcirculation, that is, before and after thrombolytic therapy in a thrombus model using the functional nano-thrombolytic agent IPN@UK, combined with label-free CM with LSMI *in vivo* (Scheme 1).Significantly, whole-course changes, including RBC activity, thrombogenesis and thrombolytic therapy, were well surveilled and assessed using synergy CM and LSMI. These results demonstrate this synergy strategy potential for early detection of thrombus and evaluation of the effect of drug thrombolysis.

## 2 Materials and methods

### 2.1 Materials

#### 2.1.1 Synthesis of iron-oxide nanoparticle

The iron-oxide nanoparticles (IPN) were prepared by our previous published method [[38], [39], [40]]. Briefly, iron-oxide nanoparticles (5 ​mg) were dissolved in chloroform and mixed with alkyl-PEI2k-PEG2k (2.5 ​mg) in chloroform solution (0.5 ​mL). Then the mixed chloroform solutions (0.5 ​mL) were dropwise added into water (5 ​mL) with continuously sonication; the IPN nanoparticles were obtained after the removal of chloroform by rotary evaporation.

The image and size of IPN nanoparticles were characterized by Transmission electron microscope (Tecnai G2 Spirit BioTwin, Holland) and Zeta sizer Nano-ZS instrument (Malvern Instruments, Malvern, UK), respectively, and the results are shown in the supplemental material (Supplementary Fig. S1, Supplementary Fig. S2).

#### 2.1.2 Conjugation of iron-oxide nanoparticle with UK

In our previous study, we confirmed that this functional nanomaterial IPN has good biocompatibility, is non-toxic, non-hazardous and easy to metabolize, and helps to improve the stability of proteins [40], making it a high-quality carrier for protein delivery.

Using 1.5 ​mg/ml of iron-oxide nanomaterials mixed with 1.0 ​mg/ml of saline dissolved urokinase solution, divided into four groups according to the ratio of nanoparticles to urokinase of 1:0.5, 1:1, 1:2.5, and 1:5, respectively, and were stirred by magnetic stirrer for about 30 ​min and left to stand at room temperature. By measuring the zeta potential of the iron-oxide nanoparticles, urokinase, and the UK Nanocomposites (IPN@UK) in four different ratios respectively, we can obtain from the information that urokinase is negatively charged at −4.9, iron-oxide nanoparticles present a positive charge of 35.33, and as the mixing ratio of materials and urokinase increases, the mixed drug prepared in the mixing ratio of magnetic nanomaterials: urokinase as 1:5 is obviously apparent as negatively charged (Supplementary Figure S2 C-D), it can be seen that the UK Nanocomposites (IPN@UK) is completely wrapped by urokinase, confirming the successful connection of the material with urokinase. IPN@UK was characterized as demonstrated in Supplementary Figs. S1–S2, as shown in TEM images, nano-size and potential information. Also, we measured the particle size change of IPN@UK at 48 ​h. It can be seen that IPN@UK has good stability at 48 ​h.

### 2.2 Animal model

#### 2.2.1 Construction of thrombosis models

This study was conducted following the rules established by the Center for Molecular Imaging and Translational Medicine at Xiamen University. The protocol was approved by the Ethics Committee for Animal Experimentation of Xiamen University.

ICR mice weighing 20 ​g–25 ​g were used for all studies. The animals are kept and used in strict compliance with the relevant laboratory regulations. A total of 15 mice were used for this study (three experimental groups of five mice each). Mice in all groups were anesthetized by continuous inhalation of isoflurane. After successful anesthesia, the mice were fixed in the supine position on a sterile thermal plate at 37 ​°C to maintain body temperature, and the lower limbs were debrided, and a 0.5 ​cm x 0.5 ​cm incision was made in the upper 1/3 of the lower limbs to bluntly separate the subcutaneous tissue and muscle and expose the femoral artery for about 0.5 ​cm. The saphenous vein (the continuation of the femoral vein in a superficial area) is gently lifted with a vascular clamp and a 0.6 ​cm ​× ​0.8 ​cm transparent membrane is placed under the vessel to separate it from the surrounding tissue.

A strip of filter paper approximately 0.5 ​cm ​× ​0.1 ​cm in size is removed, soaked in 20% concentrated ferric chloride solution for 5–10 ​min, and the soaked strip is wrapped around the vessel for 3–5 ​min. Using LSMI monitoring, blood flow was found to be reduced to image cavitation and thrombus modeling was successful. The filter paper was gently peeled off the vessel with forceps and the vessel tissue was washed with saline.

#### 2.2.2 Urokinase thrombolysis

Urokinase (BR, 50,000 u/g) was purchased from Shanghai YuanYe Biotechnology Co., Ltd, China, and the drug was diluted to a solution of 2500 u/ml with saline (CAS #7647-14-5, Sigma Company, China), (the LD50 of urokinase is 1000 u/g). When LSMI monitoring suggested thrombosis, 250 u/25 ​g units of urokinase was injected from the tail vein for thrombolysis after 20–30 ​min 2.2.3 IPN@UK thrombolysis.

The preparation method was as described above. When LSMI monitoring suggested thrombosis, 0.1 ​ml/25g (body weight) of the prepared magnetic nanoparticle-urokinase solution is injected from the tail vein 20–30 ​min later to lyse the thrombus.

### 2.3 Capillary microscopy (CM)

The schematic diagram of CM is shown in Scheme 1. The system includes a custom optical detector for imaging and a personal computer (PC) for data processing. The detector is shown in Scheme 1. It comprises four light-emitting diodes (LED) with a wavelength of 420 ​nm and a nominal power of 60 ​mW, and an image acquisition system. The image acquisition system is constituted by a two-dimensional CMOS camera (Aptina, MT9V024) with an image size of 744 ​× ​482 pixels (pixel size: 6 ​μm ​× ​6 ​μm) and a framerate of 60 ​Hz, an optical magnification system, and an objective with a numerical aperture (NA) of 0.25. The total optical magnification is 11. Two linear polarizers with orthogonal polarization directions were used to remove the scattering by the surface. The first polarizer (Polarizer1) had an annular shape and was placed in front of an annular board of LEDs, as shown in Scheme 1. The second one (Polarizer2) had a circular shape and was put in front of the object lens. A quartz glass with a thickness of 1 ​mm was placed between the objective and the mouse. The skin of mouse was covered with 50% glycerol and the refractive index was 1.395. The imaging experiment was performed 2 ​min after the application of glycerol. In the wavelength range used, the refractive index of epidermis tissue is between 1.41 and 1.49. The refractive index of glycerol differs slightly from that of the biological tissue, which reduces light scattering on the surface and improves the imaging contrast and the imaging depth. The PC was used to acquire the optical images and videos. A custom-made program was used for measuring the speed of blood flow.

### 2.4 Laser speckle microcirculation imaging (LSMI)

The system laser had a wavelength of 780 ​nm. All images were acquired by a CMOS sensor with a resolution of 1472x1104 pixels, a field of view of approximately 12x15 cm, and an imaging frame rate of 20 fps. After the mice were anesthetized and modeled (as described above), the distance between the CMOS and the mouse legs was adjusted to the optimal position for LSMI imaging.

## 3 Results and discussion

### 3.1 Optical flow diagram of single RBC migration

**Fig. 1:**
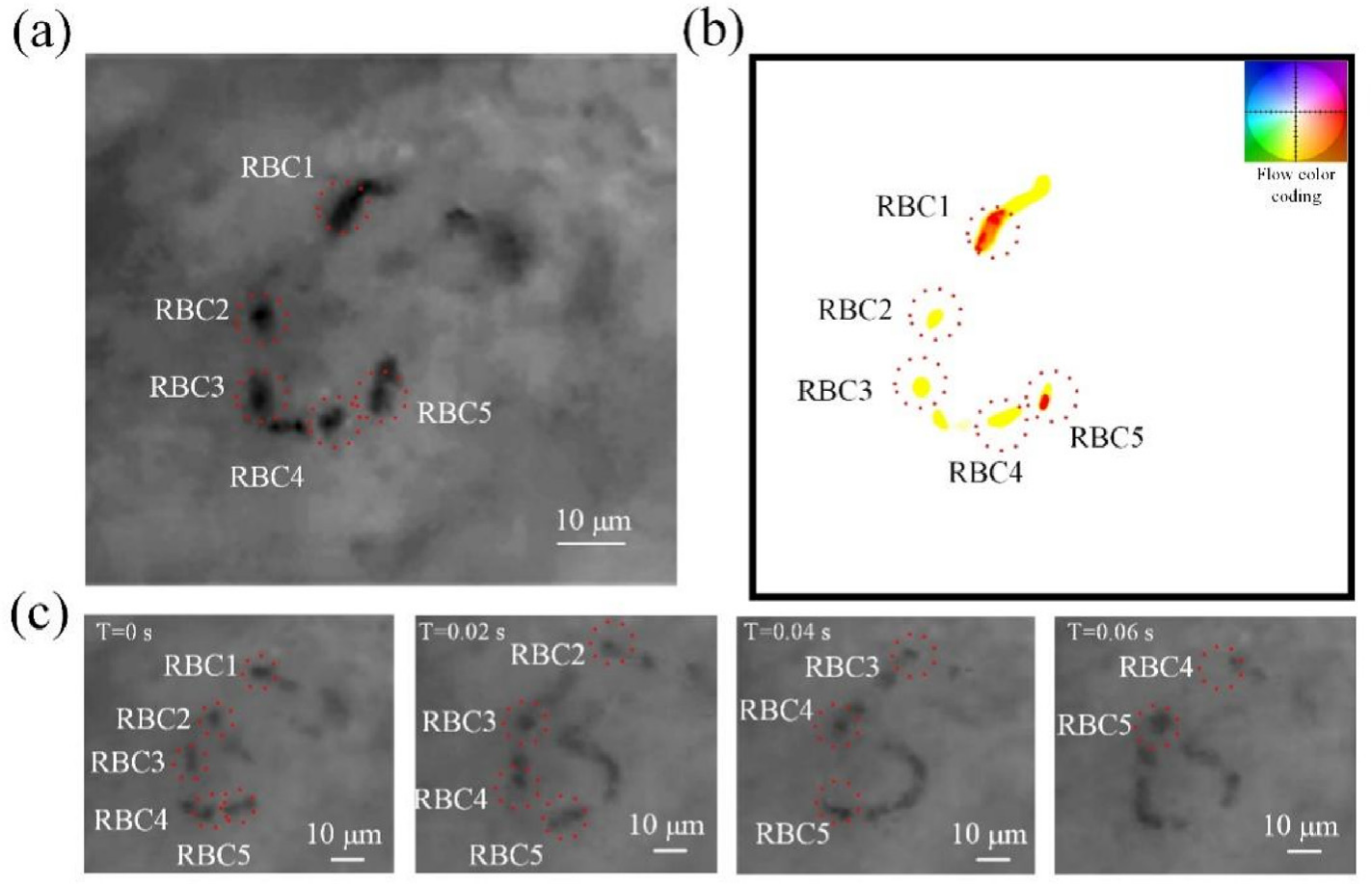
(a). A frame of dynamic microcirculation video, and single RBCs were clearly visualized. (b) Optical flow diagram of RBCs in the Fig. 1 (a). (c) Frames of the microcirculation video. Single RBCs movements were marked by red dotted circles tracked by human expert. (For interpretation of the references to color in this figure legend, the reader is referred to the Web version of this article.)

Consecutive images of microcirculation were obtained from a mouse model. A frame of the video is shown in the Fig. 1(a), and multi frame images are shown in Fig. 1(c). The time interval of frames is 1/60 ​s. Single RBCs were clearly visualized, and were marked by red dotted circles in Fig. 1(a) and (c). The optical flow diagram calculated from Fig. 1(a) is shown in Fig. 1(b) according to the methods in the literature [41,42]. As is shown in Fig. 1(a) and (b), The image and optical flow diagram of RBC1-RBC5 are corresponding. The optical flow field is that every pixel in the picture has an X-direction and Y-direction displacement, so the optical flow obtained after the above optical flow calculation is a two-dimensional image with the same size as the original image. In this work, flow color coding is used for visualization of the optical flow field, and smaller vectors are lighter, and color represents the direction, as shown in Fig. 1(b).

### 3.2 Speed measurement of blood flow in capillary by optical flow method

**Fig. 2:**
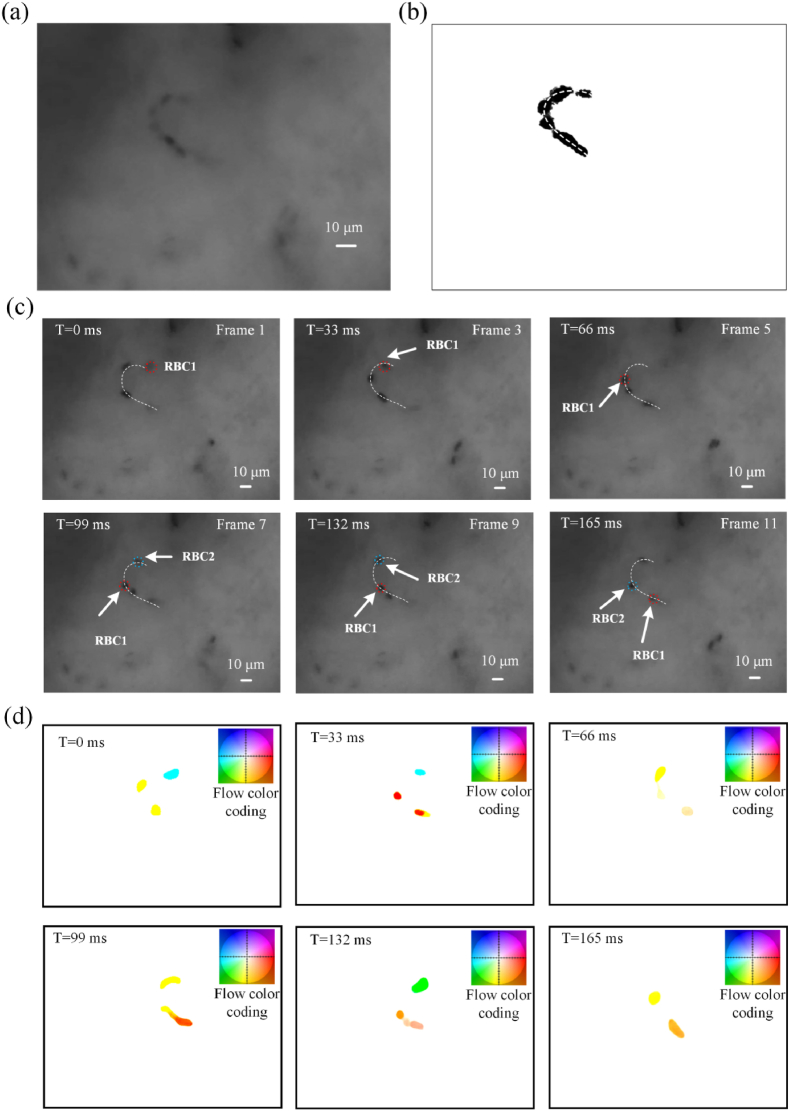
Measurement of the speed of blood flow in capillary *in vivo*. (a) A frame of dynamic microcirculation video. (b) Trajectory of RBCs obtained by multi-frame image superposition, and the skeleton curve of the trajectory is calculated as shown by dashed line. (c) Frames of the dynamic video. RBC1 and RBC2 were marked and tracked by red and blue dashed circles. (d) Optical flow diagrams of the frames in Fig. 2(c). Smaller vectors are lighter, and color represents the direction. The “ticks” on the axes of the flow color coding denote a flow unit of one-pixel. (For interpretation of the references to color in this figure legend, the reader is referred to the Web version of this article.)

Dynamic video of capillary was collected from a mouse model. A frame of the video is shown in the Fig. 2(a), and multi-frame images are shown in Fig. 2(c). The multi-frame images in Fig. 2(c) were superimposed and processed to remove the background, as shown in Fig. 2(b), which showed the migration movement trajectories of RBCs. The optical flow diagrams of the frames in Fig. 2(c) were calculated and shown in Fig. 2(d). The “ticks” on the axes of the flow color coding denote a flow unit of one-pixel in Fig. 2(b). From Fig. 2(d), the average speed of RBCs can be calculated, and the value is 0.37 ​mm/s. The speed of RBC1 and RBC2 can also be obtained by dividing the distance they move by the time of movement by human expert. Two RBCs are tracked and marked by red and blue dashed circles. In Fig. 2(c), RBC1 moves 47 ​μm in 11 frames, so its speed is 0.28 ​mm/s. RBC2 moves 25 ​μm in 5 frames, so its speed is 0.38 ​mm/s. The average speed of RBC1 and RBC2 is 0.33 ​mm/s, which can represent the blood flow speed. The average speed of RBCs calculated by optical flow method is agree well with the result of RBC tracking by human expert. This suggests that we can achieve cell-level high-resolution imaging and blood flow velocity measurements with CM imaging.

### 3.3 Monitoring the process of thrombosis

**Fig. 3:**
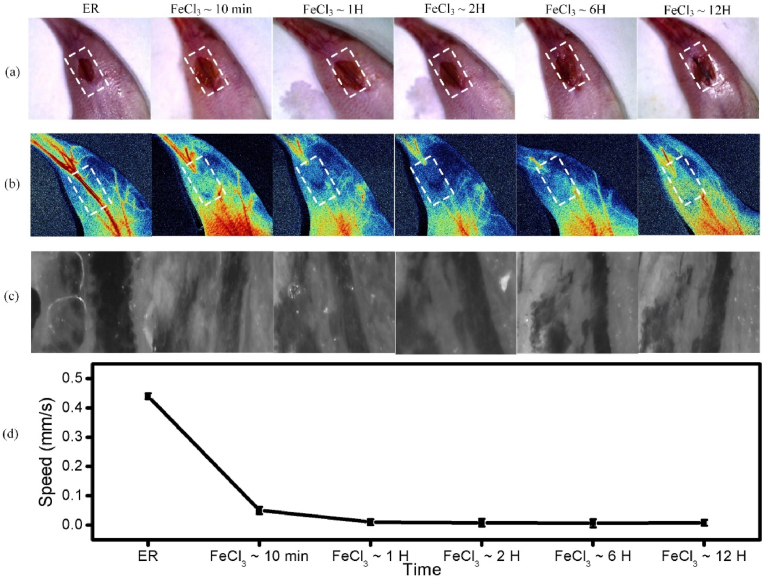
(a) Optical photographs of the thrombus model establishment in mice from the beginning of the control to the formation process at 10 ​min, 1 ​h, 2 ​h, 6 ​h, and 12 ​h. (b) LSMI images of the thrombus model establishment in mice from the beginning of the control to the formation process at 10 ​min, 1 ​h, 2 ​h, 6 ​h, and 12 ​h. (c) CM images of the thrombus model establishment in mice from the beginning of the experiment to the formation process at 10 ​min, 1 ​h, 2 ​h, 6 ​h, and 12 ​h, respectively. (d) is the blood flow speed (mm/s) at the same time point measured by the CM. The video collected by CM is in the Supplementary Videos 1-6. ER: Epidermal removal.

To observe the process of thrombus formation, we used CM and LSMI to observe the process of thrombus formation. The experimental results are shown in Fig. 3. Fig. 3(a) shows optical photographs of thrombus formation in mice with leg incisions at 10 ​min, 1 ​h, 2 ​h, 6 ​h, and 12 ​h. Fig. 3(b) shows LSMI images of thrombus formation in mice with filter paper infiltrated with FeCl_3_ solution at 10 ​min, 1 ​h, 2 ​h, 6 ​h, and 12 ​h. We can see that at 10 ​min LSMI suggests complete blockage of blood flow and little recovery by 12 ​h and the surrounding tissues at the site of vascular embolization in the legs of the mice already show more obvious damage. Meanwhile, in Fig. 3(c), CM images show that after 10 ​min administration with FeCl_3_ solution, the thrombus had formed, but some RBCs migration could be observed after 1 ​h and 2 ​h, which may be due to the vascular regulation of the mouse. LSMI can observe the state of blood flow in a large range, while CM can further observe the dynamic details of a single RBC. LSMI can observe the state of blood flow in a large range, while CM can further observe the dynamic details of a single RBC. The two imaging modalities are complementary to each other. The video collected by CM is shown in Supplementary Videos 1-6.

As can be seen from the experimental results, LSMI allows the observation of blood flow information from normal flow to the apparent process of blocked blood flow. CM imaging allows the direct observation of RBC flow and thrombus formation in the vessel. The data of blood flow velocity (mm/s) are shown in Fig. 3(d), and it can be clearly seen that the blood flow velocity continuously slows down to complete block with the formation of thrombus and does not recover within 12 ​h. We achieved high-resolution imaging of thrombus using the synergistic imaging mode of label-free CM and LSMI to image small changes in thrombus at an early stage, which facilitates the development and evaluation of thrombotic drugs.

### 3.4 Monitoring the thrombolysis with urokinase

**Fig. 4:**
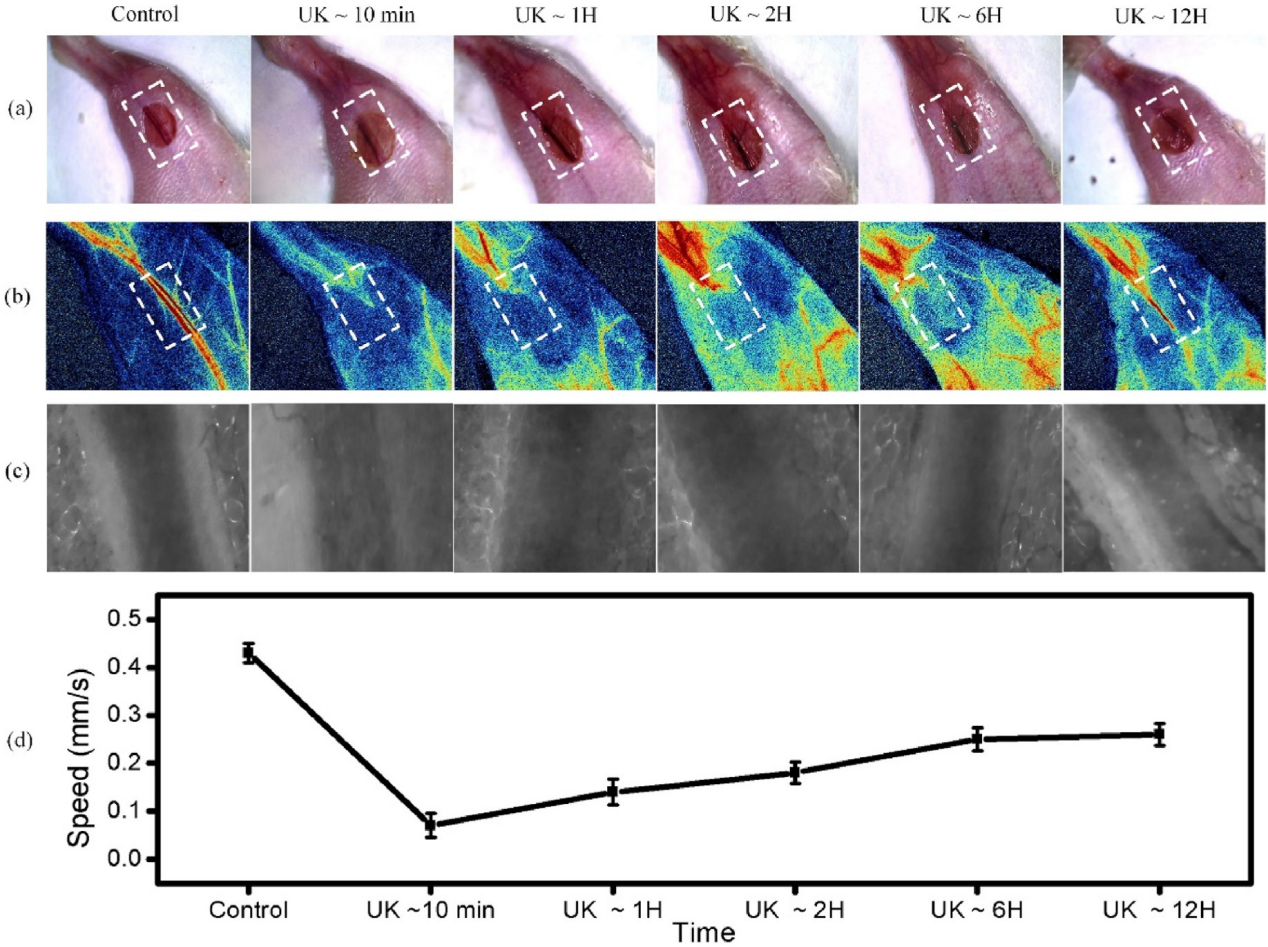
(a) Optical photograph of the process of thrombolysis at 10 ​min, 1 ​h, 2 ​h, 6 ​h, and 12 ​h after urokinase injection. (b) LSMI images of the process of thrombolysis at 10 ​min, 1 ​h, 2 ​h, 6 ​h, and 12 ​h after urokinase injection. (c) CM images of the process of thrombolysis at 10 ​min, 1 ​h, 2 ​h, 6 ​h, and 12 ​h after urokinase injection, respectively. (d) is the blood flow speed (mm/s) at the same time point measured by the CM. The video collected by CM is in the Supplementary Videos 7-12. UK: urokinase.

To verify the thrombolytic effect of urokinase, 250 u/25 ​g units of urokinase was injected from the tail vein when LSMI monitoring suggested thrombosis, and we monitored the thrombolytic process with imaging using LSMI and CM at 10 ​min, 1 ​h, 2 ​h, 6 ​h, and 12 ​h after urokinase tail vein injection. In Fig. 4, we can see that at 10 ​min after urokinase injection, the blood flow was still completely blocked, but the flow rate was slowly and gradually restored over time. At 12 ​h after urokinase injection, the LSMI images and CM videos show that the thrombus has dissolved. In conclusion, LSMI and CM imaging allowed direct observation of early thrombolysis of RBC blood flow and the whole process of thrombus lysis. In addition, although the thrombus was able to dissolve gradually by tail vein injection of urokinase, it was slower and longer, and some damage to the tissue around the thrombus in the mouse leg was already visible in the optical photographs. We finely and effectively evaluated the efficacy of the currently common thrombolytic drug urokinase using a synergy imaging mode combining label-free CM and LSMI, whose thrombolytic efficiency needs to be further improved.

The following are the supplementary data related to this article:

Video S1 2 Video S1

Video S2 3 Video S2

Video S3 4 Video S3

Video S4 5 Video S4

Video S5 6 Video S5

Video S6 7 Video S6

Supplementary video related to this article can be found at https://doi.org/10.1016/j.mtbio.2022.100408

The following are the supplementary data related to this article:

Video S7 8 Video S7

Video S8 9 Video S8

Video S9 10 Video S9

Video S10 11 Video S10

Video S11 12 Video S11

Video S12 13 Video S12

### 3.5 Monitoring the thrombolysis with IPN@UK

**Fig. 5:**
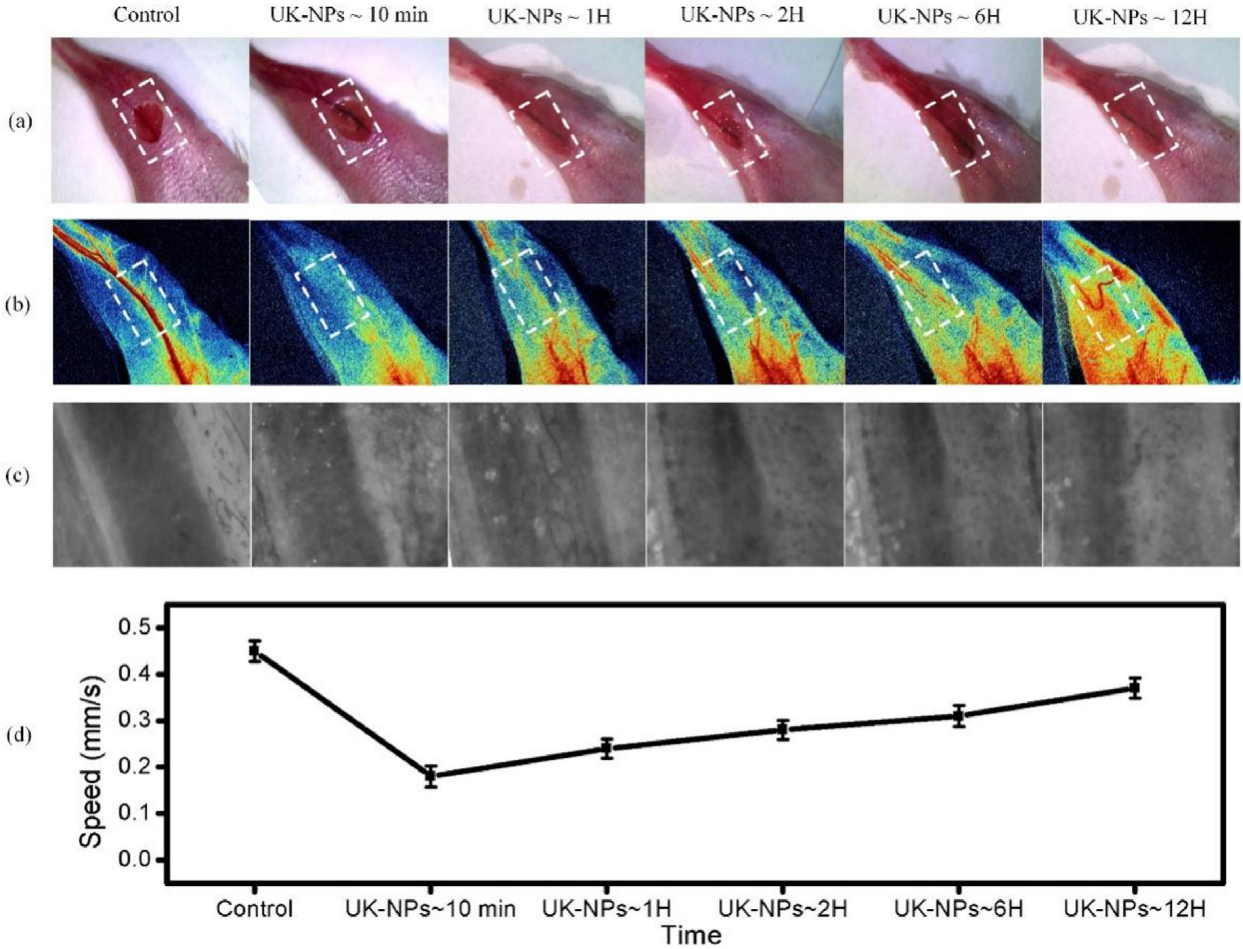
(a) Optical photograph of the process of thrombolysis at 10 ​min, 1 ​h, 2 ​h, 6 ​h, and 12 ​h after IPN@UK injection. (b) LSMI images of the process of thrombolysis at 10 ​min, 1 ​h, 2 ​h, 6 ​h, and 12 ​h after IPN@UK injection. (c) CM images of the process of thrombolysis at 10 ​min, 1 ​h, 2 ​h, 6 ​h, and 12 ​h after IPN@UK injection, respectively. (d) is the blood flow speed (mm/s) at the same time point measured by the CM. The video collected by CM is in the Supplementary Videos 13-18. UK-NPs: urokinase nanoparticles.

When LSMI monitoring suggested thrombosis, 0.1 ml/25 ​g of IPN@UK was injected from the tail vein 20–30 ​min later to lyse the thrombus. Similarly, we monitored the thrombolytic process with LSMI and CM imaging at 10 ​min, 1 ​h, 2 ​h, 6 ​h, and 12 ​h after IPN@UK tail vein injection. In Fig. 5, we can see that although the blood flow was still blocked at 10 ​min after IPN@UK tail vein injection, as in the urokinase group. However, 1 ​h after the drug injection, the blood flow rate was restored and, more importantly, the thrombus was gradually dissolved over time. Cell-level high-resolution imaging of thrombus and speed measurement of blood flow were realized with CM imaging, which can be used to finely evaluate the early process of drug thrombolysis. At the same time, LSMI modality was used to determine the status of thrombus in a large field of view and could be used to rapidly assess the effectiveness of thrombolytic drugs. From the experimental results, it can be seen that thrombolysis with IPN@UK showed a significant increase in thrombus dissolution rate compared with thrombolysis with UK only.

In Fig. 5(a), perivascular injury can be seen, we consider that this may be because of individual differences in mice and the unavoidable local soft tissue injury that may result from continuous exposure of the skin incision during the surgical manipulation of the constructed model. Considering the variability of individual mice and thus expanding the sample size, or how to exclude the interference of individual differences during the procedure to have a better consistency of results is what we will continue to study next. In our previous study [40], we investigated the biodistribution of this iron oxide nanoparticles in the liver and kidney. Mice were sacrificed at different time points and then organ tissues were harvested for ICP studies. We observed that a large percentage of IPN accumulated in the liver and spleen due to uptake by the reticuloendothelial system [43], but both decreased to control levels 24 ​h after injection, which prevented potential long-term toxicity, while accumulation in the kidney was negligible. Similarly, we confirmed that IPN contributes to the stability of the protein, allowing it to be well distributed to the site of action. In the hindlimb embolization model used in this study, biodistribution may have little effect, but this will also continue to be explored in depth in our future studies.

The following are the supplementary data related to this article:

Video S13 14 Video S13

Video S14 15 Video S14

Video S15 16 Video S15

Video S16 17 Video S16

Video S17 18 Video S17

Video S18 19 Video S17

## 4 Conclusions

In this work, a synergy imaging modality that combined label-free CM and LSMI was developed, and dynamic visualization of RBCs migration and capillary blood flow were realized. To extend the application of synergy imaging in the direction of nanoprobe molecular imaging, we constructed the IPN@UK thrombolytic drug, performed drug thrombolysis experiments and high-resolution imaging of the thrombolytic process by LSMI and CM imaging system on a mouse thrombus model, and measured the blood flow velocity, and the results verified that IPN@UK thrombolysis showed a significant improvement in thrombolysis rate compared with the use of a single urokinase thrombolytic drug. Significantly, entire process changes, including RBC activity, thrombo-genesis and thrombolytic therapy, were well surveilled and assessed. These results demonstrate the potential of this synergy imaging strategy for early detection of thrombus and assessment of the effect of drug thrombolysis, which not only provides hemodynamic information about the thrombus in a large field of view, but also provides high-resolution visualization of single cells. In conclusion, the synergy imaging of CM and LSMI can realize RBC and blood flow imaging, which is particularly important for the diagnosis of vascular related diseases and development of thrombolytic drugs.

## Credit author statement

**Fei Ye**: Conceptualization, Methodology, Formal analysis, Investigation, Writing – original draft, Visualization. **Bei Zhang**: Methodology, Investigation, Writing – review & editing. **Lige Qiu**: Conceptualization, Validation, Investigation. **Yunrui Zhang**: Investigation, Writing – review & editing. **Yang Zhang**: Conceptualization, Methodology, Supervisions. **Jian Zhang**: Methodology. **Qingliang Zhao**: Conceptualization, Methodology, Funding acquisition. **Ligong Lu**: Conceptualization, Methodology, Supervision, Funding acquisition. **Zhenlin Zhang**: Conceptualization, Methodology, Supervision, Project administration, Funding acquisition.

## Data availability statement

The data presented in this study are available on request from the corresponding author.

## Declaration of competing interest

The authors declare that they have no known competing financial interests or personal relationships that could have appeared to influence the work reported in this paper.
