# Supplementary material for: In vivo real-time red blood cell migration and microcirculation flow synergy imaging-surveyed thrombolytic therapy with iron-oxide complexes
Source: Mater Today Bio. 2022 Aug 26;16:100408. doi: 10.1016/j.mtbio.2022.100408 (PMC9463387; doi:10.1016/j.mtbio.2022.100408)
Supplement: Multimedia component 1 [file mmc1.docx]

**Supplementary Information**

*In Vivo* Real-Time Red Blood Cell Migration and Microcirculation Flow Synergy Imaging-Surveyed Thrombolytic Therapy with Iron-oxide Complexes

Fei Ye ^a, 1^, Bei Zhang ^b, 1^, Lige Qiu ^a, 1^, Yunrui Zhang ^b^, Yang Zhang ^b^, Jian Zhang ^c^, Qingliang Zhao ^b,^ ^***^, Ligong Lu^a,^ ^**^, and Zhenlin Zhang ^d, *^

^a^ Zhuhai Interventional Medical Center, Zhuhai Precision Medical Center, Zhuhai People’s Hospital (Zhuhai Hospital Affiliated With Jinan University), Zhuhai, 519000, P.R. China

^b^ State Key Laboratory of Molecular Vaccinology and Molecular Diagnostics, Center for Molecular Imaging and Translational Medicine, School of Public Health, Shenzhen Research Institute of Xiamen University, Xiamen University, Xiamen, 361102, P.R. China

^c^ The Sixth Affiliated Hospital of Guangzhou Medical University, Qingyuan People’s Hospital, Department of Biomedical Engineering, School of Basic Medical Sciences, Guangzhou Medical University, Guangzhou, 511436, P.R. China

^d^ Department of Pharmacy, Zhuhai People’s Hospital (Zhuhai Hospital Affiliated With Jinan University), Zhuhai, 519000, P.R. China

**^*^**  Corresponding author

**^**^** Corresponding author

**^***^** Corresponding author

E-mail addresses: [jnudoctor@163.com](mailto:jnudoctor@163.com) (Z. Zhang), [luligong1969@jnu.edu.cn](mailto:luligong1969@jnu.edu.cn) (L. Lu), [zhaoql@xmu.edu.cn](mailto:zhaoql@xmu.edu.cn) (Q. Zhao).

^1^ These authors contributed equally to this work.

**Abstract:** Nanotherapeutics as a nascent method has attracted widely interest on the treatment of thrombosis. However, due to the limited temporal and spatial resolution of conventional imaging modalities, the dynamic visualization the thrombogenesis and evaluation of the effect of thrombolytic drugs are facing severely difficulties in vivo. In addition, the development of high targeting, short circulation time, and small size thrombolysis nanotherapeutics agents requires further research. Herein, we report a synergy imaging modality that combining a label-free capillary microscopy and laser speckle microcirculation imaging, which realized dynamic visualization of single red blood cell migration and large-field dynamic blood flow. In this work, we investigated the red blood cells migration and blood flow velocity response before and after treated through introducing a functional nano-thrombolytics, iron-oxide complexes coated urokinase (IPN@UK) on an orthotopic animal model in vivo. The functionalized IPN@UK nanocomposites exhibited outstanding thrombolysis effect. Significantly, whole-course changes, including red blood cell activity, complex thrombolytic therapeutics, were well surveilled and evaluated using dual-modality combining imaging strategy. These results show this synergy imaging strategy not only can achieve multiscale non-invasive visualization of dynamic thrombus events in real-time, but also can quantify hemodynamics information of thrombus. Our study demonstrates the potential of this synergy imaging method, which for early detection of thrombus, evaluation of the effect of drug thrombolysis, developing the thrombolytic drugs, and imaging-guide thrombolytic therapy in living systems.

**Keywords:** Thrombosis; Thrombolysis; Red blood cell; Capillary microscopy; Microcirculation flow imaging


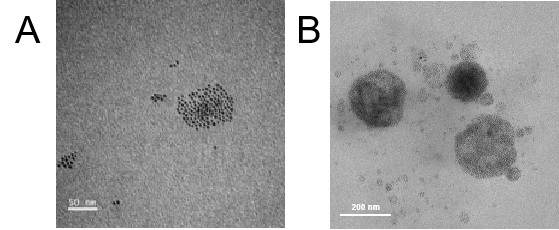


**Figure S1**. (A) TEM images of IPN nanoparticles, scale bar: 50 nm (B) TEM images of IPN@UK, scale bar: 200 nm


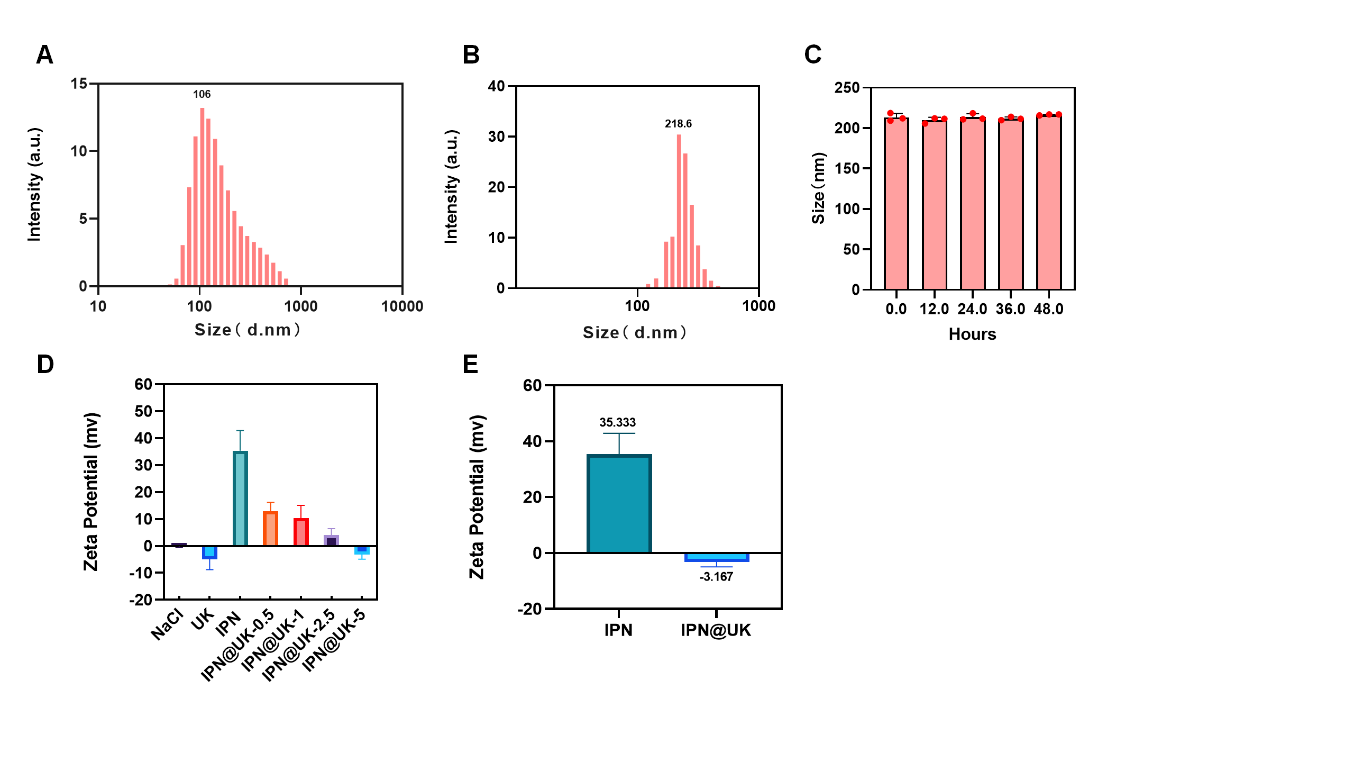


**Figure S2**. IPN and IPN@UK characterization (A) Diameter distribution of IPN（B）Diameter distribution of IPN@UK**.** (C) Particle size stability of IPN@UK for 48hours. (D-E) Zeta potential of IPN and IPN@UK. IPN nanoparticles present a positive charge of 35.333, and as the mixing ratio of materials and urokinase increases, the mixed drug prepared in the mixing ratio of magnetic nanomaterials: urokinase as 1:5 is obviously apparent as negatively charged, it can be seen that the UK Nanocomposites (IPN@UK) is completely wrapped by urokinase, confirming the successful connection of the material with urokinase.

**Supplementary Video 1.** A video of dynamic microcirculation after removal of the epidermis captured using CM.

**Supplementary Video 2.** A video of dynamic microcirculation after 10 minutes of applying ferric chloride captured using CM.

**Supplementary Video 3.** A video of dynamic microcirculation after 1 hour of applying ferric chloride captured using CM.

**Supplementary Video 4.** A video of dynamic microcirculation after 2 hours of applying ferric chloride captured using CM.

**Supplementary Video 5.** A video of dynamic microcirculation after 6 hours of applying ferric chloride captured using CM.

**Supplementary Video 6.** A video of dynamic microcirculation after 12 hours of applying ferric chloride captured using CM.

**Supplementary Video 7.** A video of dynamic microcirculation in the urokinase thrombolysis group taken using CM after removal of the epidermis.

**Supplementary Video 8.** In the urokinase thrombolysis group, dynamic microcirculation video taken using CM at 10 minutes after urokinase injection.

**Supplementary Video 9.** In the urokinase thrombolysis group, dynamic microcirculation video taken using CM at 1 hour after urokinase injection.

**Supplementary Video10.** In the urokinase thrombolysis group, dynamic microcirculation video taken using CM at 2 hours after urokinase injection.

**Supplementary Video 11.** In the urokinase thrombolysis group, dynamic microcirculation video taken using CM at 6 hours after urokinase injection.

**Supplementary Video 12.** In the urokinase thrombolysis group, dynamic microcirculation video taken using CM at 12 hours after urokinase injection.

**Supplementary Video 13.** A video of dynamic microcirculation in the IPN@UK thrombolysis group taken using CM after removal of the epidermis.

**Supplementary Video 14.** In the IPN@UK thrombolysis group, dynamic microcirculation video taken using CM at 10 minutes after IPN@UK injection.

**Supplementary Video 15.** In the IPN@UK thrombolysis group, dynamic microcirculation video taken using CM at 1 hour after IPN@UK injection.

**Supplementary Video 16.** In the IPN@UK thrombolysis group, dynamic microcirculation video taken using CM at 2 hours after IPN@UK injection.

**Supplementary Video 17.** In the IPN@UK thrombolysis group, dynamic microcirculation video taken using CM at 6 hours after IPN@UK injection.

**Supplementary Video 18.** In the IPN@UK thrombolysis group, dynamic microcirculation video taken using CM at 12 hours after IPN@UK injection.
